# Diabetic Inhibition of Preconditioning- and Postconditioning-Mediated Myocardial Protection against Ischemia/Reperfusion Injury

**DOI:** 10.1155/2012/198048

**Published:** 2011-08-01

**Authors:** Xia Yin, Yang Zheng, Xujie Zhai, Xin Zhao, Lu Cai

**Affiliations:** ^1^The Cardiovascular Center, The First Hospital of Jilin University, 71 Xinmin Street, Changchun 130021, China; ^2^KCHRI, The Department of Pediatrics, University of Louisville, Louisville, KY 40202, USA; ^3^Breast Surgery, China-Japan Union Hospital of Jilin University, Changchun 130033, China

## Abstract

Ischemic preconditioning (IPC) or postconditioning (Ipost) is proved to efficiently prevent ischemia/reperfusion injuries. Mortality of diabetic patients with acute myocardial infarction was found to be 2–6 folds higher than that of non-diabetic patients with same myocardial infarction, which may be in part due to diabetic inhibition of IPC- and Ipost-mediated protective mechanisms. Both IPC- and Ipost-mediated myocardial protection is predominantly mediated by stimulating PI3K/Akt and associated GSK-3*β* pathway while diabetes-mediated pathogenic effects are found to be mediated by inhibiting PI3K/Akt and associated GSK-3*β* pathway. Therefore, this review briefly introduced the general features of IPC- and Ipost-mediated myocardial protection and the general pathogenic effects of diabetes on the myocardium. We have collected experimental evidence that indicates the diabetic inhibition of IPC- and Ipost-mediated myocardial protection. Increasing evidence implies that diabetic inhibition of IPC- and Ipost-mediated myocardial protection may be mediated by inhibiting PI3K/Akt and associated GSK-3*β* pathway. Therefore any strategy to activate PI3K/Akt and associated GSK-3*β* pathway to release the diabetic inhibition of both IPC and Ipost-mediated myocardial protection may provide the protective effect against ischemia/reperfusion injuries.

## 1. Introduction

Acute myocardial infarction (AMI) is a worldwide problem that threatens the human's health both in the developed and developing countries. AMI is often induced by the complete thrombotic occlusion of coronary arteries at the site of a ruptured atherosclerotic plaque. Prompt reperfusion is a definitive treatment to salvage ischemic myocardium from inevitable death. Experimental and clinical investigations suggest that although reperfusion can salvage the ischemic myocardium, it can also induce side effect, called as ischemia/reperfusion injuries. It is appreciated now that lethal myocardial injury caused by ischemia/reperfusion accounts for up to 50% of the final infarct size of a myocardial infarct [1].

Myocardial ischemia/reperfusion injury is a complex pathophysiological event, resulting in serious acute and chronic myocardial damage. It is characterized by a cascade of acutely initiated local inflammatory responses, metabolic disorder, and cell death, leading to myocardial ultrastructural changes and remodeling and subsequently myocardial systolic and diastolic dysfunction [2–4]. Myocardial ischemia/reperfusion injury also induces ventricular arrhythmias, resulting in circulation collapse and sudden death [5, 6].

Numerous studies have demonstrated that inflammation following ischemia/reperfusion injury exacerbates myocardial injury [4, 7]. In addition to inflammation, profound alterations in myocardial metabolism, such as the disarrangement of glycolysis and fatty acid oxidation, also significantly impact on the cell integrity and functional recovery of the myocardium [8]. Evidence from previous studies suggests that reactive oxygen or nitrogen species (ROS or RNS), including superoxide radicals, hydrogen peroxide, hydroxyl radicals, singlet oxygen, nitric oxide, and peroxynitrite play major contribution to myocardial ischemia/reperfusion injury [9, 10]. These ROS and/or RNSs, which are formed within the ischemic myocardial cells and in the first few moments of reperfusion, are known to be cytotoxic to surrounding cells. In addition, it is also widely accepted that apoptotic cell death is involved in the development of ischemic myocardial damage [11]. Therefore, how to protect the ischemic myocardium from reperfusion injury is the key issue for cardiologist and cardiovascular physicians. This review briefly overviews the status of ischemic preconditioning (IPC) and ischemic postconditioning (Ipost) with an emphasis of the diabetic effects on the myocardial protection of IPC and Ipost as well as possible mechanisms.

## 2. Ischemic Preconditioning, Postconditioning, and Their Myocardial Protective Mechanisms

### 2.1. Ischemic Preconditioning and Its Myocardial Protection

**Figure 1:**
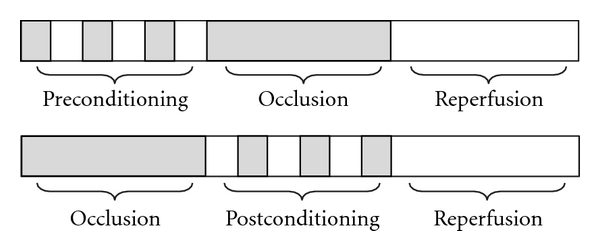
The illustration of IPC and Ipost. IPC means that transient and repeat ischemia and reperfusions were given before the occlusion of the coronary artery. Ipost means that transient and repeat ischemia and reperfusions were given after the occlusion and before the reperfusion of coronary artery.

Murry et al. (1986) first found the potent myocardial protection by preconditioning the ischemic myocardium when they gave transient and repeat ischemia and reperfusion before the occlusion of the coronary artery in dog heart [12]. They found that multiple brief ischemic episodes actually protected the myocardium from a subsequent sustained ischemic insult. They called this protective effect as IPC (Figure 1). IPC is a well-described adaptive response by which brief exposure to ischemia/reperfusion before sustained ischemia markedly enhances the ability of the myocardium to withstand a subsequent ischemic insult [13]. The protection of IPC is displayed as the reduction of ischemia/reperfusion-induced infarct size, arrhythmia, and the improvement of contractile and diastolic function of the myocardial muscle. Consequently, many studies indicated that IPC was an endogenous protection for AMI, by inducing phosphatidylinositol 3-kinase (PI3K), protein kinase C (PKC) and JAK/STAT pathways [12, 14–17]. Among these, the activation of PI3K/protein kinase B (Akt) pathway was found to play an important role in protecting myocardial ischemia/reperfusion injury [15, 16, 18]. The PI3K/Akt pathway affects cell survival by a variety of substrates, including apoptotic proteins, endothelial nitric oxide synthase (eNOS), and PKC [19, 20]. More recent interest has focused on glycogen synthase kinase-3*β* (GSK-3*β*) as a distal kinase, phosphorylated (and hence inactivated) by other kinases, including Akt and p42/p44 MAPK/ERK [21, 22]. GSK-3*β* is a multifunctional Ser/Thr kinase that plays important roles in necrosis and apoptosis of cardiomyocytes. GSK-3 activity has been associated with many cell processes, including the regulation of multiple transcription factors, the Wnt pathway, nuclear factor *κ*B, endoplasmic reticulum stress, embryogenesis, apoptosis and cell survival, cell cycle progression, cell migration, and so on [23, 24].

IPC produces myocardial protection by phosphorylating and consequently inactivating GSK-3*β* [21]. However, since ischemic event is unpredictable and IPC is also invasive, myocardial protection by IPC is difficult to be used in clinics. In this review, we do not introduce the detail status of IPC myocardial protection and possible mechanisms since these issues have been extensively discussed in a few recent excellent reviews [25–28].

### 2.2. Ischemic Postconditioning and Its Myocardial Protection

The Ipost came into notice of Zhao et al. (2003) when they moved the transient and repeat ischemia/reperfusions to after the occlusion and before the reperfusion, as illustrated in Figure 1 [2]. Subsequently, a lot of researchers reported the same protective effects [29, 30]. They found that cycles of brief reperfusion and ischemia performed immediately at the onset of reperfusion following a prolonged ischemic insult markedly limited reperfusion injury. Like IPC, the Ipost is also a powerful approach to protect the ischemic myocardium from reperfusion-induced damage [31–33]. In clinics, with the development of percutaneous coronary intervention emerged as an exciting innovative treatment strategy, it makes Ipost possible to intervene AMI. A recent analysis of data on infarct size and ischemic zone size indicates that current reperfusion therapy salvages more than 50% of the ischemic myocardium in approximately half of the patients with AMI [34].

It has been supported by several studies that Ipost protected the myocardium against the detrimental effects of lethal myocardial reperfusion injury by limiting oxidative stress, reducing calcium accumulation, maintaining endothelial function, and reducing inflammation [35–37]. Subsequent studies have identified a number of signaling pathways which are activated by Ipost, and involve in the myocardial protection of Ipost. Among these pathways, reperfusion injury salvage kinase (RISK) pathway was the first signaling cascade to be linked to Ipost [21], which showed that Ipost was capable of recruiting prosurvival signal cascades including PI3K/Akt, PKC, GSK-3*β*, eNOS, and guanylyl-cyclase, as disclosed for the mechanisms of IPC myocardial protection (see the above discussion).

**Figure 2:**
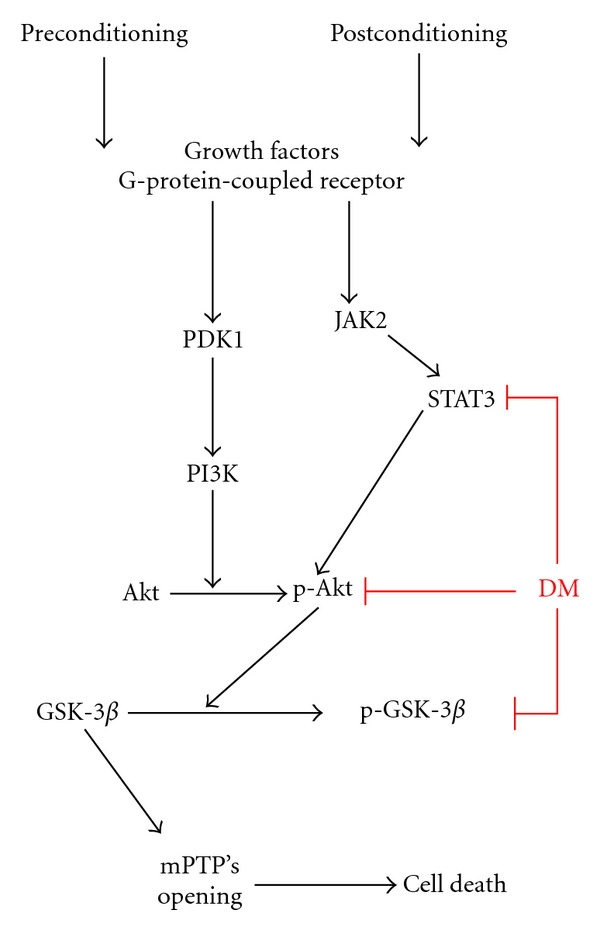
Major signaling pathways of IPC- and Ipost-mediated protection against cardiac cell death. Myocardial protection of IPC and Ipost were proposed to be mediated by stimulation of the prosurvival signaling pathway—PI3K/Akt pathway to inhibit the GSK-3*β* activation either via PI3K pathway or JAK2/STAT3 pathway. Diabetes (DM) can inhibit the activation of STAT3 or Akt to consequently activate GSK-3*β* that in turn induces mitochondrial cell death that is the critical cellular event for ischemia/reperfusion-induced myocardial infarction.

The discovery of IPC and Ipost, including pharmacological preconditioning and postconditioning, as the two major forms of endogenously protective mechanisms in the myocardium have encouraged us to explore new ways to protect the myocardium from ischemia/reperfusion and have enriched our knowledge of the molecular basis of injury and survival during ischemia/reperfusion [13]. In both IPC- and Ipost-mediated myocardial protections, PI3K/Akt activation is considered as an initial step that induces phosphorylation of downstream kinases to inhibit the several pro-apoptotic factors and mitochondrial permeability transition pore (mPTP)'s opening at reperfusion, as illustrated in Figure 2 [23, 38–44]. One of the downstream targets of the RISK pathway is GSK-3*β* that plays important roles in necrosis and apoptosis of cardiomyocytes [23]. GSK-3*β* links to the regulation of a variety of cellular functions including glycogen metabolism, gene expression, and cellular survival. Experimental studies have demonstrated that the phosphorylation or inactivation of GSK-3*β* confers myocardial protective effects through its potential mitochondrial effects that include the inhibition of mPTP's opening and the control of mitochondrial adenine nucleotide transport through the outer mitochondrial membrane [35–37]. The mPTP is a nonselective large conductance channel in the mitochondrial inner membrane, which is physiologically closed. The mPTP remains closed during ischemia but opens at the onset of reperfusion [45], and modulation of the mPTP opening at early reperfusion can protect the myocardium from reperfusion injury [46, 47].

Opening of mPTPs is involved in cell death induced by a variety of causes, including ischemia/reperfusion, alcohol, endotoxin, and anticancer agents [48]. In addition to Ca^2+^, ROS and/or RNS-caused accumulation of inorganic phosphate and depletion of ATP all can open mPTPs [49, 50]. It is also clear that all of these mPTP opening stimuli are induced in cardiomyocytes subjected to long-sustained ischemia/reperfusion. Ipost significantly elevated the threshold of mPTP's opening in myocardial mitochondria [23]. The inhibition of mPTP's opening plays a critical end effector for the myocardial protective effects of Ipost. Juhaszova et al. [21] first reported that GSK-3*β* activity is a determinant of the threshold for mPTP's opening in cardiomyocytes. Therefore, GSK-3*β* plays a critical role in IPC- and Ipost-mediated myocardial protection.

So far, there were two studies that have examined the role of GSK-3*β* as an obligatory mediator of Ipost using transgenic mice and showed different results. Gomez et al. [35] found that mice containing a mutant form of GSK-3*β* (which cannot be phosphorylated and inhibited) were resistant to the myocardial infarct-limiting effects of Ipost in situ, suggesting that GSK-3*β* inactivation is required for Ipost's myocardial protection. Contrast to the study of Gomez et al., Nishino et al. [51] have reported that mice with a mutant form of both GSK-3*β* and GSK-3*α* in which the Akt phosphorylation sites were changed, thereby rendering them to resistant to inactivation, were still amenable to the myocardial infarct-limiting effects of both IPC and Ipost. This study suggests that GSK-3*β* and GSK-3*α* inactivation are not necessary for myocardial protection in these settings. Therefore, the exact role of GSK-3*β* in the setting of Ipost remains further investigation, particularly under different conditions.

## 3. Diabetic Inhibition of Ischemic Preconditioning- and Postconditioning-Mediated Myocardial Protection against Ischemia/Reperfusion Injury

Epidemiological data show that diabetes is a major risk for cardiovascular morbidity and mortality [52, 53]. Coronary artery diseases leading to myocardial infarction and myocardium failure are one of the major chronic complications of diabetes, accounting for >75% of hospitalizations in diabetic patients. The mortality rate of diabetic patients after AMI is 2–6 folds higher than that of nondiabetic patients [54, 55]. Increased mortality or increased myocardial injury following AMI in diabetes is thought probably because of the high prevalence of other risk factors, that is, hypertension, hyperlipidemia, and advanced coronary artery diseases [56]. The poor prognosis may be also in part because of an increase in the myocardial injury in response to ischemia and reperfusion [57].

It is well known that insulin regulates metabolism in the myocardium by modulating glucose transport, glycolysis, glycogen synthesis, lipid metabolism, protein synthesis, growth, contractility, and apoptosis in cardiomyocytes [58, 59]. Myocardial insulin resistance develops in animal models of both type 1 and type 2 diabetes [59]. These insulin-stimulated effects have been shown to be reduced in the myocardium and cardiomyocytes of diabetic rats [60], which may be the main reason for the increase in myocardial injury in response to ischemia and reperfusion in diabetic subjects.

In normal physiological status, insulin can regulate the metabolism of glucose through PI3K/Akt pathway. Insulin binds to its receptor and phosphorylates insulin receptor's substrates (IRS) such as IRS protein 1–4, Shc, Grb-2 associated binder-1, and APS adapter protein. These substrates have the SH2 structural domain and can provide the orientation sites for other signaling protein molecules, including the downstream signaling molecules of PI3K [61, 62]. Activated PI3K can phosphorylate the PI's substrates specifically to produce PIP2 and PIP3. PIP1 and PIP2 can translocate the PI3K-dependent kinase (PDK1) and Akt from the cytoplasm to plasma membrane. Under these conditions, Akt can be phosphorylated at Thr308 and Ser473, and the activated Akt then phosphorylates GSK-3*β*. The phosphorylation of GSK-3*β* inactivates its activity, which will release its inhibition of the synthesis of glycogen, as shown in Figure 2. The activity of GSK-3*β* is two-fold higher in diabetes than that of nondiabetes. Hyperglycemia and hyperinsulinemia can both activate the GSK-3*β* [43, 44, 63]. The activated GSK-3*β* can inhibit the myocardial transduction of insulin signaling and the utilization of glucose through the phosphorylation of IRS-1.

We have recently reported for the first time that the activation of GSK-3*β* played the pivotal role in diabetes-induced energy disarrangement and consequently pathological remodeling in the myocardium [63]. This study suggests that the activation of GSK-3*β* plays an important role in the development of diabetic cardiomyopathy. 

Diabetes is an independent risk factor for ischemic myocardium disease; therefore, whether diabetes could decrease the IPC and/or Ipost protection against ischemia/reperfusion-induced myocardial damage has been questioned. Tosaki et al. found that IPC did not afford protection against ischemic damage in diabetic subjects [76]. Other studies also showed that STZ-induced diabetes significantly aggravated myocardial ischemia/reperfusion injury and blunted the protective effects of IPC [77, 78]. However, whether diabetes abrogates IPC- or Ipost-mediated myocardial protection depends on IPC times or the periods of diabetes. For instance, Tsang et al. [15] discovered that in normal Wistar rats, one, two, and three cycles of IPC significantly reduced infarct size induced by ischemia/reperfusion; however, in diabetic Goto-Kakizaki (GK) rats, only three cycles of IPC reduced infarct size induced by ischemia/reperfusion, compared with GK control hearts. Both one and two cycles of IPC failed to afford reductive effect on the infarct, suggesting that the diabetic heart has a high threshed to IPC stimulus-induced myocardial protection. In addition, Shi-Ting et al. [79] also showed that mice with diabetes for 4 weeks showed a tolerance to ischemia/reperfusion-induced damage as compared to normal rats; IPC of these diabetic mice remained affording partial myocardial protection. In contrast, mice with diabetes for 8 weeks showed a low tolerance to ischemia/reperfusion damage as compared to normal mice, and the IPC-induced myocardial protection was not evident. These findings suggest that short-term diabetes makes the myocardium more tolerant, like an adaptive response, but long-term diabetes makes the myocardium more susceptible to ischemia/reperfusion-induced damage, like a decompensated response.

Recently, Przyklenk et al. [80] have assessed the consequences of a major risk factor—diabetes on the infarct-sparing effect of stuttered reflow using type 1 and type 2 diabetic mouse models. They gave the isolated buffer-perfused myocardium for 30 min ischemia, and the myocardium received either standard reperfusion or three to six 10s cycles of stuttered reflow as Ipost. They found that Ipost-reduced infarct size via upregulation of extracellular signal-regulated kinase 1/2 (ERK1/2) in normoglycemic mice, but diabetic myocardium was refractory to Ipost-induced cardioprotection. They also found that in the type-1 diabetic model, Ipost's protective effects were reversed by the restoration of normoglycemia. Therefore, this study provided strong evidence for a profound, but potentially reversible, defect induced by diabetes in the myocardial protection of Ipost. In a study by Drenger et al. [81], however, the protective effects of Ipost were found to be inhibited in the diabetes rats, and the diabetic inhibition of Ipost's myocardial protection was not relieved by insulin-induced normoglycemia. The discrepancy between these two studies may be also due to hyperglycemic times; as the hyperglycemic time increases, the inhibited protective function of Ipost by diabetes may become irreversible.

As the myocardial protection of IPC and Ipost is mediated by a number of signaling pathways, the blunted myocardial protection mediated by IPC in diabetes may be related to the impairment in myocardial protective signaling pathways such as the PI3K/Akt pathway, as illustrated in Figure 2 [2, 15, 77]. Since signal transducer and activator of transcription (STAT) 3-mediated signaling pathway has been found to play an important role in the cardiac protection induced by IPC [14] and Ipost [69]. Downregulation of STAT3 was found to be a causative of abolishment of the cardiac protection mediated by IPC [17] and Ipost [81–83] under several conditions. Therefore, STAT3 downregulation may be one of the mechanisms for diabetic inhibition of Ipost-mediated cardiac protection, as discussed by Drenger et al. [81]. Reportedly erythropoietin (EPO) has an IPC-like effect to show myocardial protection against ischemia/reperfusion-induced damage [73, 74]. However, Ghaboura et al. have shown the attenuation of EPO-mediated myocardial protection under diabetic condition [43].

## 4. Diabetic Activation of GSK-3***β*** Plays a Critical Role in Diminishing IPC- and Ipost-Mediated Myocardial Protective Function

In the above sections, we mentioned that there are several signaling pathways that may involve in the myocardial protection mediated by IPC or Ipost. As shown in Figure 2, however, inactivation of GSK-3*β* has been considered as the pivotal step for both IPC and Ipost's myocardial protection. Furthermore, studies have demonstrated that diabetes-induced activation of GSK-3*β* and impairment of RISK play critical roles in diabetes-induced myocardial oxidative damage and remodeling [43, 63]; other studies also reported that the activity of GSK-3*β* is twice in diabetic patients compared to that of nondiabetic patients [84]. Therefore, whether diabetic activation of GSK-3*β* blunts IPC and Ipost's myocardial protection really needs to be investigated [83, 85, 86].

To date, studies have demonstrated a decreased protective effect of IPC on AMI in diabetic subjects [43, 44, 83, 85–87]. It is clear that IPC produces myocardial protection by phosphorylation of GSK-3*β* that inhibited the opening of mPTP, but the activity of GSK-3*β* was found to be elevated during diabetes [21, 23, 35, 63]. Yadav et al. [44] investigated the role of GSK-3*β* in attenuating the cardioprotective effect of IPC using a type-1diabetic rat model. They found that IPC had protective effect on normal rat myocardium, but this cardioprotective effect of IPC was significantly attenuated in diabetic rat. At the same time, they found that GSK-3*β* inhibitors, including lithium chloride, indirubin-3 monooxime, and SB216763, significantly reduced the myocardial damage and decreased infarct size in diabetic rat myocardium. This study suggests that diabetes-induced attenuation of myocardial protection mediated by IPC involves in the activation of GSK-3*β*. In addition, Ghaboura et al. [43] also demonstrated that the attenuation of EPO-mediated myocardial protection from ischemia/reperfusion under diabetic condition was related to the decrease in EPO-stimulated GSK-3*β* phosphorylation. The administration of GSK-3*β* inhibitor SB216763 protected the hearts from ischemia/reperfusion-induced damage in control and diabetic groups [43]. Therefore, the inhibition of IPC myocardial protection in the diabetes is most likely related to the activation of GSK-3*β* [43, 44].

Because Ipost and IPC share some common signal transduction cascades proposed above (Figure 2), which include the activation of survival protein kinase pathways [13]. In the study by Drenger et al. [81], they demonstrated that diabetes can impair the protective effect of Ipost on myocardial damage or infarction through inhibition of STAT 3-mediated PI3K/Akt pathways. Up to now, there remains no proof to indicate that diabetes can inhibit the protective effect of Ipost on the myocardium; therefore, it remains to be further explored.

## 5. Is It Possible to Prevent the Diabetic Inhibition of IPC or Ipost Myocardial Protection against Ischemia/Reperfusion Injury?

We have demonstrated that diabetes-induced myocardial oxidative damage and inflammation mainly due to the activation of GSK-3*β*. When we inactivated GSK-3*β* activity with its inactivator in diabetic mice, diabetes-induced myocardial damage were almost completely prevented [63]. In addition, we have discussed above that inactivation of GSK-3*β* with its specific inactivators can also directly afford the myocardial protection in diabetic animals treated with GSK-3*β* inactivators [43, 44]. Therefore, any reagents that can inactivate GSK-3*β* may have the potential to be applied for the prevention of diabetic inhibition of IPC- and/or Ipost-mediated myocardial protection. Except for the consideration of GSK-3*β* inhibitors as discussed above and also listed in the Table 1, the following reagents (Table 1) may also have such potential.

Zinc (Zn) is an interesting candidate because Zn is an important trace element found in most body tissues as bivalent cations and has essential roles in human health. Zn has also an insulin-like function that was found also to be related to its inactivation of GSK-3*β* [88]. We have demonstrated that Zn supplementation to diabetic mice could significantly prevent the development of myocardial oxidative damage, remodeling, and dysfunction in these diabetic mice [64]. Although we did not explore whether the myocardial protection by Zn supplementation in these diabetic mice is mediated by the inactivation of GSK-3*β* by supplied Zn, other studies have reported that Zn also inactivated GSK-3*β* in several conditions. In the experiment from Chanoit et al. [42], for instance, they found that the treatment of myocardial H9c2 cells with ZnCl_2 _(10 *μ*M) for 20 min significantly enhanced GSK-3*β* phosphorylation at Ser9, indicating that exogenous Zn can inactivate GSK-3*β* in H9c2 cells. Other experiments [41] also demonstrated that Zn also increased mitochondrial GSK-3*β* phosphorylation. This may indicate an involvement of the mitochondria in the action of Zn.

Zn applied at reperfusion period reduced cell death in the cells subjected to ischemia/reperfusion, which confirmed that Zn may act as an inactivator of GSK-3*β* to provide a myocardial protection at reperfusion [41, 42, 89]. Besides the direct inactivation of GSK-3*β*, Zn was also reported to stimulate Akt phosphorylation by inhibiting Akt negative regulators, including phosphatase and tensin homologue on chromosome 10 (PTEN) and protein tyrosine phosphatase 1B (PTP1B) [65–67]. Inactivation of PTEN and/or PTP1B may also contribute to Zn's inactivation of GSK-3*β* via Akt activation [41]. Therefore, Zn may inhibit GSK-3*β* by direct and indirect mechanisms to protect the myocardium from diabetic activation of GSK-3*β*-mediated pathogenic effects.

In addition to Zn protective effects, other substrates are also reported to exert their protective effect, as IPC and Ipost, on ischemia/reperfusion-induced cardiac damage. For instance, adenosine leads to the activation and/or translocation of PKC, PI3K, and mitogen-activated protein kinase (MAPK) and, subsequently, affords IPC- or Ipost-like myocardial protection at the level of mitochondrial targets [68]. Endogenous opioids have also been documented to be involved in protective effects of Ipost [69, 70]. The administration of EPO at the time of reperfusion afforded a beneficial effect on Ipost myocardial protection in rabbits [90] and mice [73]. EPO administration just prior to reperfusion has reduced infarct size in isolated rat and dog hearts, and even in canine hearts. Furthermore, EPO administration even 5 min after the reperfusion has also provided protective responses [74, 91]. Lamont et al. reported that both melatonin and resveratrol, as found in red wine, protected the myocardium in an experimental model from myocardial infarction via the survivor activating factor enhancement pathway [92]. Fang et al. demonstrate that sevoflurane administered immediately during early reperfusion prevented the myocardial infarction [75].

Although all these substances can afford myocardial protective effects on ischemia/reperfusion in the models without diabetes, whether these substances can modify diabetic individuals to maintain the myocardial protection of IPC and Ipost remains to be explored in the future studies.

## 6. Conclusion

Epidemiological data show that diabetes is a major risk for cardiovascular diseases and the mortality of diabetic patients with acute myocardial infarction is 2–6 folds higher than that of nondiabetic patients with the same myocardial infarction. The poor prognosis may be at least in part because of diabetic inhibition of IPC- and Ipost-mediated protective mechanisms against ischemia/reperfusion injuries. Emerging evidence indicates that both IPC- and Ipost-mediated myocardial protection predominantly be mediated by stimulating PI3K/Akt and associated GSK-3*β* pathway while diabetes-mediated pathogenic effects are found to be mediated by inhibiting PI3K/Akt and associated GSK-3*β* pathway. Therefore, diabetic inhibition of IPC- and Ipost-mediated myocardial protection may be mediated by the activation of GSK-3*β* pathway, which suggests a possibility that we may activate PI3K/Akt indirectly to inactivate GSK-3*β* pathway or use GSK-3*β* inactivator directly to inactive GSK-3*β* pathway to preserve IPC- and/or Ipost-mediated myocardial protection under diabetic conditions. Although there is not enough experimental and epidemiological evidence to support our assumption, it was worthy to be explored in the future studies.

## Figures and Tables

**Table 1:** Potential candidates that may have protective effect against ischemia reperfusion injury related with Akt/GSK-3*β* pathway.

Potential candidates	Target of signaling pathway	Reference
Lithium chloride	GSK-3*β* inhibitor	[44]
Indirubin-3 monooxime	GSK-3*β* inhibitor	[44]
SB216763	GSK-3*β* inhibitor	[44]
Zinc	Inactivation of GSK-3*β* directly or indirectly	[42, 64–67]
Adenosine	Activation/translocation of PKC, PI3K, and MAPK	[68]
Endogenous opioids	JAK-STAT pathway and then inactivation of GSK-3*β*	[69–72]
Erythropoietin	Activate Akt and inhibit GSK-3*β*	[43, 73, 74]
Sevoflurane	Phosphorylates Akt and then GSK-3*β*	[75]

Except for the GSK-3*β* inhibitors, most of other potential candidates may exert their protective effect against ischemia reperfusion injury through activation of Akt and then inactivation of GSK-3*β*.
